# Genome-Wide Analysis of *Snf2* Gene Family Reveals Potential Role in Regulation of Spike Development in Barley

**DOI:** 10.3390/ijms24010457

**Published:** 2022-12-27

**Authors:** Gang Chen, Kohei Mishina, Hongjing Zhu, Shinji Kikuchi, Hidenori Sassa, Youko Oono, Takao Komatsuda

**Affiliations:** 1 Institute of Crop Science, National Agriculture and Food Research Organization (NARO), 2-1-2 Kannondai, Tsukuba 305-8602, Japan; 2 Graduate School of Horticulture, Chiba University, 648 Matsudo, Matsudo 271-8510, Japan; 3 Crop Research Institute, Shandong Academy of Agricultural Sciences/National Engineering Research Center of Wheat and Maize/Shandong Technology Innovation Center of Wheat, Jinan 252100, China

**Keywords:** *Hordeum vulgare*, ATP-dependent chromatin remodeling, evolution, expression analysis, spike development

## Abstract

Sucrose nonfermenting 2 (Snf2) family proteins, as the catalytic core of ATP-dependent chromatin remodeling complexes, play important roles in nuclear processes as diverse as DNA replication, transcriptional regulation, and DNA repair and recombination. The *Snf2* gene family has been characterized in several plant species; some of its members regulate flower development in Arabidopsis. However, little is known about the members of the family in barley (*Hordeum vulgare*). Here, 38 *Snf2* genes unevenly distributed among seven chromosomes were identified from the barley (cv. Morex) genome. Phylogenetic analysis categorized them into 18 subfamilies. They contained combinations of 21 domains and consisted of 3 to 34 exons. Evolution analysis revealed that segmental duplication contributed predominantly to the expansion of the family in barley, and the duplicated gene pairs have undergone purifying selection. About eight hundred *Snf2* family genes were identified from 20 barley accessions, ranging from 38 to 41 genes in each. Most of these genes were subjected to purification selection during barley domestication. Most were expressed abundantly during spike development. This study provides a comprehensive characterization of barley *Snf2* family members, which should help to improve our understanding of their potential regulatory roles in barley spike development.

## 1. Introduction

In eukaryotes, genetic information is packaged into nucleosomes, which are repetitive structures consisting of 147-bp DNA segments wrapped around a central histone octamer composed of two copies each of the core histones H2A, H2B, H3, and H4 [1]. Nucleosomes are further compacted to form the fundamental unit of higher order organization, called chromatin, to fit into the nucleus. Such compaction of nucleosomes in chromatin restricts accessibility of the wrapped DNA to regulatory proteins such as transcription factors [2]. Two chromatin-remodeling enzymes have been implicated in making the packaged DNA accessible. One alters the contact between the histone octamer and the DNA by utilizing the energy derived from ATP hydrolysis [3,4]. The other modulates the specific residues of DNA and histones through adding or removing covalent modifications such as methylation, acetylation, phosphorylation, and ubiquitylation [5]. Proteins involved in the two processes are referred to as chromatin remodelers and modifiers, respectively. Both types are usually associated with other proteins in different multi-subunit complexes, but also are frequently involved in the same multi-subunit complexes. These multi-subunit complexes are also called chromatin remodeling complexes [6].

Sucrose nonfermenting 2 (Snf2) family proteins harbor a catalytic ATPase region, and are homologous to the Snf2 protein that was first identified from yeast ATP-dependent chromatin remodeling complexes [7]. Snf2 family members are classified into six groups on the basis of the similarity within the ATPase region, namely Snf2-like, Swr1-like, Rad54-like, Rad5/16-like, SSO1653-like, and SMARCAL1-like [8,9]. Each group can be further subdivided into several subfamilies that are conserved from yeasts to animals and plants [4]. More than 1300 proteins in the Snf2 family from bacteria, yeast, *Drosophila*, mouse, human, and *Arabidopsis thaliana* are divided into 24 distinct subfamilies [8]. The Arabidopsis genome has 41 *Snf2* family genes, rice has 40, and tomato has 45 [9,10,11].

Snf2 proteins play vital roles in plant growth and development [12,13,14,15], as well as in biotic and abiotic stresses [16]. Flower development is a complex process that is crucial for reproduction and grain yield. Several Arabidopsis Snf2 proteins are involved in flowering time [17,18], inflorescence architecture [19,20], and floral organ identity [21]. Rice Snf2 family members have been identified [10]. Little is known, however, about the family in other grass species, and the role of Snf2 proteins in grass flower development remains obscure.

Barely (*Hordeum vulgare*) is one of the earliest cultivated crops; it is widely adapted to diverse environments and plays an essential role in global food production. Cultivated barley (*H. vulgare* subsp. *vulgare*) was domesticated from wild barley (*H. vulgare* subsp. *spontaneum*) approximately 10,000 years ago in the Fertile Crescent region [22,23]. Barley is an annual diploid inbreeding species, able to generate fully fertile progeny without reproductive barriers [22]. It is an ideal genomic model for the Triticeae tribe owing to its relatively simple genome and has been well studied in genomics over the past decade. The chromosome-scale genome assembly of barley cultivar Morex has been published [24] and subsequently updated to generate several versions by means of high-throughput sequencing and assembly technologies [25,26]. A pan-genome assembly with genetic variations among wild and cultivated barley accessions was released recently [27]. Wild barley is a rich resource for genetic diversity. Several genome assemblies from wild barley are now publicly available [28,29]. These data enable the identification of gene families at the genome-wide level in barley. However, the *Snf2* gene family remains largely unknown in the Triticeae. The objectives of this study were to identify the *Snf2* family members in barley and to explore potential regulatory roles in barley spike development.

## 2. Results

### 2.1. Identification of Snf2 Family Genes in Barley

**Figure 1:**
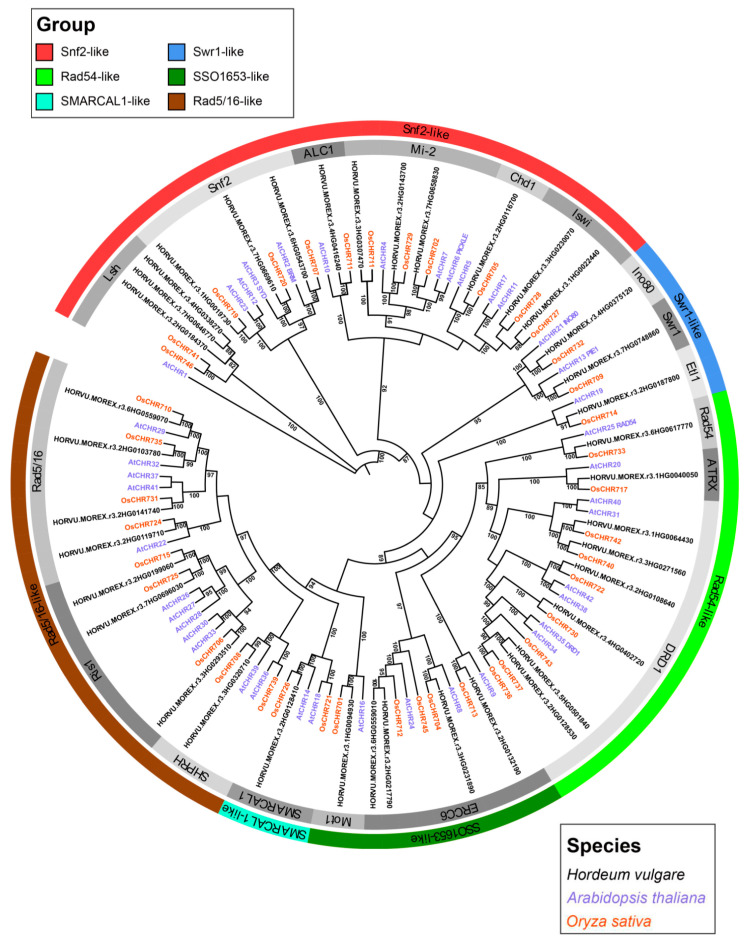
Phylogenetic tree of Snf2 proteins from barley, Arabidopsis, and rice. The maximum likelihood tree was constructed in IQ-TREE2 software with 1000 bootstrap replications. Numbers at each branch node indicate bootstrap probabilities of >80%. Different groups of proteins are highlighted with different colors. The grouping of the Snf2 family is based on the classification cited from [9].

The hidden Markov model (HMM)-based approach retrieved 39 high-confidence genes encoding putative proteins with conserved SNF2_N and Helicase_C domains from the Morex reference genome [26], of which 38 were identified as members of the barley *Snf2* family (Table 1). The nucleotide sequence of *HORVU.MOREX.r3.7HG0646770* is identical to that of *HORVU.MOREX.r3.UnG0786740*, which was assigned to chromosome unknown (chrUn) in the Morex genome, and was therefore removed. A phylogenetic tree was constructed from Snf2 proteins from barley, Arabidopsis (AtCHR) [9], and rice (OsCHR) [10] to reveal the family in barley (Figure 1). The barley Snf2 proteins were clustered into 6 groups and 18 subfamilies (Figure 1). They were distributed in all subfamilies, with varying numbers of proteins in each. Most were enriched within the Lsh, Snf2, Mi-2, Iswi, DRD1, ERRC6, Ris1, and Rad5/16 subfamilies, of which DRD1 is the largest cluster, with 6 members. In contrast, ALC1, Chd1, Ino80, Swr1, Etl1, Rad54, ATRX, Mot1, SMARCAL1, and SHPRH had 1 member each. Phylogenetic analysis identified 18 orthologous groups in 14 subfamilies across the three species, suggesting that these proteins originated from a common ancestor before the divergence of the dicots and monocots. In addition, three pairs of paralogous proteins were found in the Lsh (*HORVU.MOREX.r3.4HG0338270* and *HORVU.MOREX.r3.7HG0646770*), DRD1 (*HORVU.MOREX.r3.2HG0128530* and *HORVU.MOREX.r3.5HG0501840*), and ERRC6 (*HORVU.MOREX.r3.2HG0217790* and *HORVU.MOREX.r3.6HG0559010*) subfamilies (Figure 1), indicating that the *Snf2* family underwent gene duplication events after barley’s divergence from rice.

### 2.2. Barley Snf2 Protein Properties and Domain Organization

**Figure 2:**
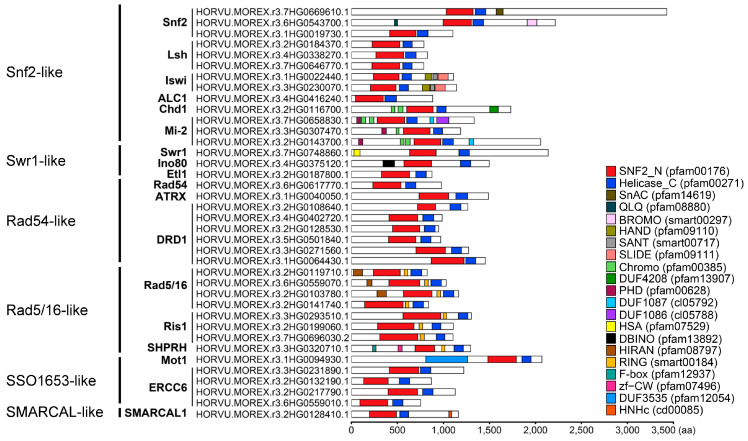
Domain architecture of barley Snf2 family proteins. Different domains are represented by bars with different colors. The scale represents the length of deduced protein sequences, all in proportion.

The amino acid length ranged between 681 and 3440 residues (Table S1). The molecular weights varied from 77 to 371 kDa, and the theoretical isoelectric point (pI) value ranged from 4.83 to 8.88. All proteins had negative grand average of hydrophobicity (GRAVY) values, varying from −0.89 to −0.22, indicating their hydrophilic nature. Prediction of subcellular localization revealed that all barley Snf2 proteins were localized in the nucleus (Table S1). There were 21 domains (Figure 2). The Chromo domain was present in the Chd1 and Mi-2 subfamilies of Snf2-like, and the RING domain was found in all subfamilies of Rad5/16. By contrast, several domains were unique to a subfamily: the HAND, SANT, and SLIDE domains were found only in Iswi, the PHD domain only in Mi-2, and the HIRAN domain only in Rad5/16.

### 2.3. Gene Structure of Barley Snf2 Family

**Figure 3:**
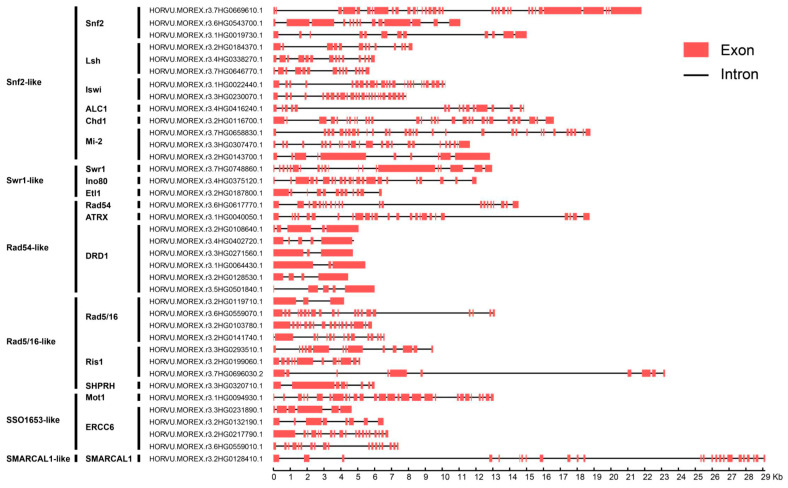
Exon–intron organization of barley Snf2 genes.

The barley Snf2 family genes had 2 to 33 introns (Figure 3). The 41 AtCHR genes had 2 to 33 introns, and 45 tomato CHR genes (SlCHR) had 1 to 37 [11]. The Snf2 subfamily had the most exons (34), and 3 genes in the DRD1 and Rad5/16 subfamilies had the fewest (3). The exon and intron lengths varied among genes, even in the same subfamily. Seventeen of the 38 barley Snf2 genes spanned >10 kb from start to stop codons, possibly attributable introns longer than 5 kb (Figure 3). Three genes in the Snf2, Ris1 and SMARCAL1 subfamilies had genomic sequences of >20 kb, with 33, 9, and 23 introns, respectively.

### 2.4. Chromosomal Distribution and Duplication Analysis of Barley Snf2 Gene Family

**Figure 4:**
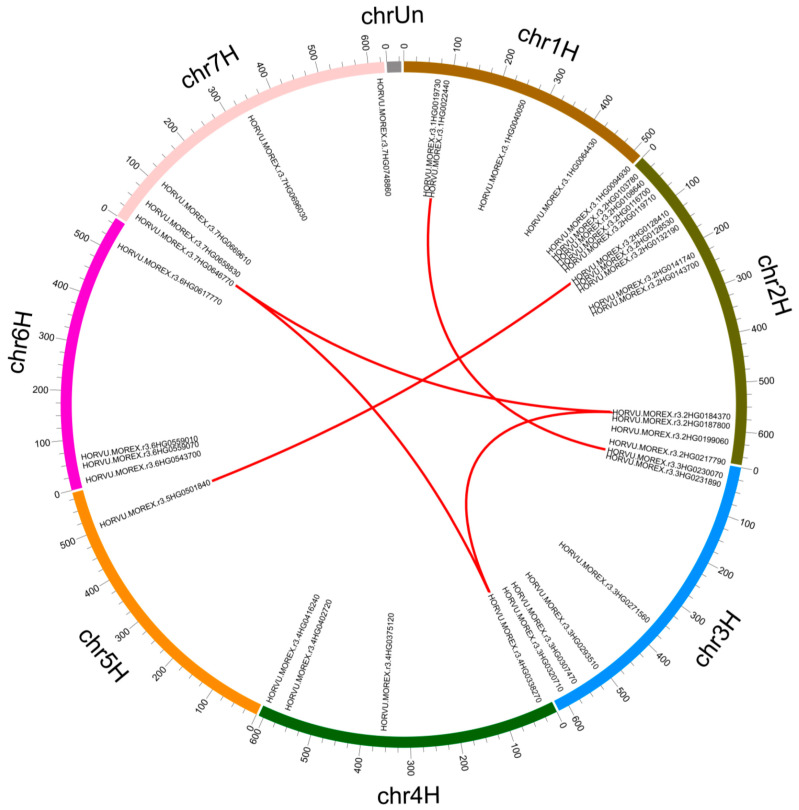
Chromosomal distribution of barley Snf2 family genes and gene duplications. Segmentally duplicated gene pairs are connected by red curves.

Chromosome localization results showed that the 38 Snf2 genes were unevenly distributed across the 7 chromosomes of the Morex genome (Figure 4). Chromosome 2H had the most genes (13), followed by chromosome 3H (6). Chromosomes 1H and 7H both had 5 genes, 4H and 6H had 4, and 5H had only 1 gene. Most Snf2 genes were located in the terminal regions of the chromosomes, with few in the central regions. Five pairs composed of 7 genes were predicted to have undergone segmental duplication, but no tandem duplication was found (Figure 4). Likewise, only segmental duplication events were found in Snf2 family genes of Arabidopsis and rice (Table S2). Selection pressure determined divergence of barley Snf2 genes after duplication. All 5 segmental-duplication gene pairs had ratios of non-synonymous (Ka) to synonymous (Ks) substitutions (Ka/Ks) lower than 1 (Table S3), suggesting that purifying selection pressure followed duplication.

### 2.5. Genetic Variation and Evolutionary Analysis of Snf2 Genes in Barley Populations

We investigated copy-number variations of Snf2 genes within subfamilies among 20 barley accessions (Table 2). About eight hundred Snf2 family genes were identified from cultivated and wild accessions, with 38 to 41 genes in each. All cultivated accessions had 38 to 41 genes, and 2 wild accessions had 40 genes. Multiple alignment analysis of Snf2 family members revealed that there are no reliable variations in copy number of this gene family across different barley accessions (Table S4). Members missing in some accessions may result from genome assembly and gene annotation since all of them without gene IDs were validated to have alignments in genomes.

To evaluate the genetic diversity of barley Snf2 genes, we also analyzed the single nucleotide polymorphisms (SNPs) in barley accessions. A total of 1342 SNPs were found in coding sequences of genes from 18 subfamilies (Table S5). Among them, 594 SNPs were detected in ERRC6 subfamily, followed by DRD1 (106) and Snf2 (105) subfamilies. By contrast, Ino80 subfamily exhibited the lowest variation with only one SNP. Further, 367 SNPs were predicted as missense variants and one was a stop lost and splice region variant (Table S6), which may affect the protein functions. In addition, phylogeny and population structure analysis of SNPs revealed distinct evolutionary divergence among different barley accessions (Figure S1). We further investigated the effect of domestication on Snf2 family evolution in the wild barley accession OUH602 and Morex, and found 38 orthologous gene pairs between the two (Figure S2). Pairwise Ka/Ks ratios of orthologous genes showed that 27 pairs were purified-selection pairs and 2 were positive-selection pairs (Table S7). These results indicate that most Snf2 genes had gone through purification selection during barley domestication.

### 2.6. Expression Analysis of Barley Snf2 Genes in Various Tissues and Different Stresses

**Figure 5:**
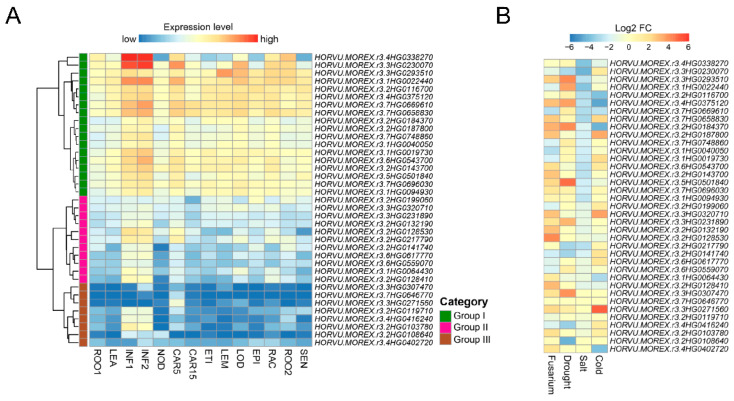
Expression analysis of barley Snf2 genes. (**A**) Transcriptional profiles of *Snf2* genes expressed in different tissues at different developmental stages in Morex. Gene expression level is shown on a graded color scale based on log_2_(FPKM + 1) values. ROO1, roots from seedlings at 17 days after planting (dap); LEA, leaves from seedlings at 17 dap; INF1, developing inflorescences of 5 mm at 30 dap; INF2, developing inflorescences of 1.0–1.5 cm at 50 dap; NOD, third stem internodes at 42 dap; CAR5, developing grains at 5 days after anthesis; CAR15, developing grains at 15 days after anthesis; ETI, etiolated leaves at 10 dap; LEM, lemma isolated from inflorescences at 42 dap; LOD, lodicule isolated from inflorescences at 42 dap; EPI, epidermal strips isolated from leaves at 28 dap; RAC, rachis isolated from inflorescences at 35 dap; ROO2, roots at 28 dap; SEN, senescing leaves at 56 dap. (**B**) Transcriptional profiles of *Snf2* genes in response to biotic and abiotic stresses. Transcript levels of each gene were detected in spike under Fusarium infection, young inflorescence under drought treatment, roots under salt treatment and leaves under cold treatment. Expression is presented as log_2_ fold change (log_2_ FC) of the mean FPKM between treatment and control. Multiple timepoints and tissue zones in each dataset were presented as mean FPKM values. Genes are in the same orders as in the hierarchical clustering of tissue expression patterns in panel (**A**).

Expression patterns of barley *Snf2* genes were analyzed by using 14 public transcriptomes from diverse tissues at different developmental stages (Figure 5A); 37 of the 38 genes were expressed with an average FPKM > 1 in at least one organ, but 1 had no data because its corresponding gene is absent in the Morex genome [25] (Table S8). Most *Snf2* genes were expressed more strongly in developing inflorescences (INF1 and INF2) than in the other organs. These expressed genes were classified into I, II, and III groups based on their expression patterns among all organs. The expression level of the Group I genes was moderate or higher than that of the Group II and III genes, most of which was moderate in only INF1, INF2, and developing grain (CAR5), and low in the other organs. Several genes were expressed predominantly in developing floral organs. *HORVU.MOREX.r3.3HG0293510* from the Ris1 subfamily was highly expressed in lemma (LEM) and lodicule tissues. The expression of 2 genes from the Iswi subfamily—*HORVU.MOREX.r3.1HG0022440* and *HORVU.MOREX.r3.3HG0230070*—was abundant in lodicule.

We also analyzed the expression profiles of barley Snf2 genes responding to biotic and abiotic stresses based on public RNA-seq datasets (Table S9). Results showed that the vast majority of genes exhibited expression changes under at least one of four stress treatments (Figure 5B). Most Snf2 genes were induced in spike in response to Fusarium infection, as well as in young inflorescence under drought stress. In contrast, more genes were suppressed by salt or cold stresses. Remarkably, almost all the Snf2 genes were repressed in root under high-salinity treatment. Furthermore, several genes showed multiple stresses-responsive changes in their expression. For example, *HORVU.MOREX.r3.3HG0230070* was suppressed by Fusarium disease, drought, and salt stresses and induced by cold stress. *HORVU.MOREX.r3.4HG0375120* was induced by Fusarium and drought stresses and repressed by salt and cold stresses. *HORVU.MOREX.r3.7HG0669610* was induced only by drought stress and repressed by the other three stresses. *HORVU.MOREX.r3.2HG0217790* showed decreased expression under both drought and salt stresses and *HORVU.MOREX.r3.3HG0271560* was induced in response to Fusarium, drought, and cold stresses.

## 3. Discussion

### 3.1. Snf2 Gene Family Shows Evolutionary Conservation in Plants

Snf2 proteins, as the core ‘motor’, have been characterized at the genome-wide level in Arabidopsis [9], tomato [11], rice [10], and sorghum [30]. Here, we identified 38 genes encoding Snf2 proteins in barley, the same as the number identified in sorghum, but fewer than in Arabidopsis (41), tomato (45), and rice (40), indicating that species and genome size differences are not directly related to the number of *Snf2* family genes.

Phylogenetic analysis using the Snf2 proteins from barley, Arabidopsis, and rice divided the 38 barley *Snf2* family genes into 18 subfamilies, highly consistent with the classification in Arabidopsis [9] and rice [10]. Intriguingly, the *Snf2* genes in 14 subfamilies displayed a 1:1:1 orthologous pattern across these species, implying that genes in these subfamilies are more highly conserved than the genes in the other subfamilies. Gene duplication is crucial in genome evolution [31,32]. We identified only five barley gene pairs that were involved in segment duplication events, as seen similarly in Arabidopsis and rice. Notably, *CHR11* and *CHR17*, in the Iswi subfamily of Arabidopsis, were identified as a segmental duplication pair here. Previous studies showed that these two genes function redundantly [33,34]. Their barley homologs were also subjected to segmental duplication, which implies that they might also function redundantly in barley, as reported in rice [10]. Moreover, the other Arabidopsis segmental duplication pair, *CHR12* and *CHR23*, has also been reported to have functional redundancy [35]. We note that these two genes correspond to a single gene in barley and rice, respectively. This implies that functional redundancy of the two Arabidopsis genes may be largely due to a segmental duplication event after dicots diverged from monocots. Two rice genes—*CHR741* and *CHR746*, which experienced segmental duplication—have three homologs in barley that also experienced segmental duplication, demonstrating that segmental duplication driving Snf2 family expansion continued after barley diverged from rice. These results indicate that segmental duplication was the main evolutionary contributor to expansion and functional diversification of the Snf2 gene family after the divergence of dicots and monocots.

Evolution of novel gene functions is a consequence of the interaction between duplication and selection. Our analysis of the barley Snf2 family showed that all segmental duplication pairs underwent purifying selection, which reduces genetic diversity [36], implying that the functional divergence of these duplicated genes might tend to be conservative. A recent study reported that purifying selection contributed to the functional redundancy of the auxin response factor (ARF) family in *Setaria italica* [37]. Therefore, we hypothesize that purifying selection restricts differentiation of Snf2 gene functions. Our results support this hypothesis since a duplicated barley gene pair is likely to function redundantly and subsequently undergoes purifying selection. Furthermore, most Snf2 genes have also experienced purifying selection during barley domestication. This indicates that the Snf2 family is highly conserved in barley evolution. Taken together, these results show that the barley Snf2 gene family shows evolutionary conservation.

### 3.2. Characteristics of Barley Snf2 Family Genes

ATP-dependent chromatin remodeling in yeast and humans is involved in nuclear processes as diverse as DNA replication [38], transcriptional regulation [39,40], DNA repair [41], and homologous recombination [42]. Here, all barley Snf2 members were predicted to localize to the nucleus, which corresponds to the reported roles of Snf2 proteins in various DNA events. This implies that several functions of Snf2 proteins are conserved between barley and other species.

Catalytic ATPase is essential for the chromatin-remodeling activity of Snf2 proteins. Catalytic ATPase is composed of SNF2_N and Helicase_C domains, which are conserved in plants [8,9]. Both domains contain ATP-binding sites: SNF2_N mediates ATP hydrolysis and Helicase_C contributes to ATP-dependent DNA or RNA unwinding. Here, barley Snf2 family members had combinations of 21 domains, in addition to two typical domains, and had diverse domains in different subfamilies. One Snf2 subfamily member carries a bromodomain, which is suggested to recognize acetyl-lysine residues on histone tails [43]. Two Iswi subfamily members harbor HAND, SANT, and SLIDE domains, which are responsible for regulation of nucleosome spacing via interacting with the linker DNA [44]. Additionally, the Chromo domain (CHROMO) has binding activity for DNA and histone [45,46]. One Chd1 subfamily member and three Mi-2 subfamily members had a Chromo domain. The implication is that functions of barley Snf2 genes are diverse among subfamilies.

### 3.3. Snf2 Family Genes May Play Regulatory Roles in Barley Spike Development

Gene expression profiles provide hints to the elucidation of potential functions. The expression patterns of barley Snf2 family members in different tissues and developmental stages revealed that most genes were abundantly expressed in developing spikes, suggesting that Snf2 family genes activate cell division. Several Snf2 genes in Arabidopsis regulate flower development processes involved in floral transition [17,18], inflorescence architecture [19,20], and floral organ identity [21]. Seven barley genes were highly expressed during spike development and had diverse expression profiles in spike developmental stages in wild barley OUH602, implying multiple roles in regulating spike development. For instance, a barley Lsh gene (*HORVU.MOREX.r3.4HG0338270*) had extremely high expression during spike development, indicating that it may contribute to the differentiation of barley spikes. A barley Mi-2 gene (*HORVU.MOREX.r3.7HG0658830*) was consistently expressed during spike development. Its Arabidopsis ortholog *CHR6/PICKLE* (*PKL*) is involved in developmental phase transition and meristem maintenance [47,48], with the implication that it is required for floral meristem induction in barley. A barley ortholog of Arabidopsis *BRM* (*HORVU.MOREX.r3.6HG0543700*) was gradually increased during spike development. *BRM* has been implicated in the regulation of flowering time [17,49], inflorescence architecture [19], and floral organ development [21], indicating that its barley ortholog may participate in regulation of floral phase transition and of inflorescence and floral meristem development. Two other barley genes from the Iswi subfamily, which were generated by a segmental duplication identified here, had highly similar expression profiles during spike development. Their Arabidopsis homologs—*CHR11* and *CHR17*—have redundant roles in flowering induction and floral organ identity [33,34,50]. A barley Iswi gene (*HORVU.MOREX.r3.3HG0230070*) had higher expression levels during spike development than another Iswi gene (*HORVU.MOREX.r3.1HG0022440*). We suggest therefore that both barley genes may be sub-functionalized for regulation of spike development. Overall, these results give insight into potential regulatory roles of Snf2 family genes involved in barley spike development. Moreover, the present study revealed that a subset of barley Snf2 genes displayed stresses-responsive changes in expression. The mutant of *Arabidopsis PKL*, *pkl*, is hypersensitive to cold stress [51], its barley homolog *HORVU.MOREX.r3.7HG0658830* was induced by cold stress. *OsCHR710*, a Rad5/16 subfamily gene, was upregulated under drought stress [52]. The barley ortholog of *OsCHR710* (*HORVU.MOREX.r3.6HG0559070*) was induced by drought stress. Thus, Snf2 genes may also play key roles on stress responses in barley.

## 4. Materials and Methods

### 4.1. Identification of Snf2 Gene Family in Barley

High-confidence protein sequences of the barley Morex genome assembly [26] were download from GrainGenes (https://wheat.pw.usda.gov/GG3/ (accessed on 25 May 2022)) and used as a local protein database. Sequences of SNF2_N (Pfam accession number PF00176) and Helicase_C (PF00271), as typical domains of Snf2 family proteins, were downloaded from the Pfam database (https://pfam.xfam.org/ (accessed on 14 July 2022)). The Hidden Markov model (HMM) profiles of these two domains were used as queries against the barley local protein database by the *hmmsearch* tool provided in HMMER v. 3.3.2 software [53] with an E-value threshold of 1e-5. After removal of short and redundant sequences, the candidate protein sequences were further examined and validated in Pfam v. 33.1 with SNF2_N (pfam00176; cl37620) and Helicase_C (pfam00271) domains in the NCBI’s Conserved Domain Database (CDD) platform (http://www.ncbi.nlm.nih.gov/Structure/cdd/wrpsb.cgi/ (accessed on 26 July 2022); [54]). Only proteins having both SNF2_N and Helicase_C domains were selected. The *Snf2* family members were also identified as above from the barley pan-genome [27] and the wild barley OUH602 genome [29].

### 4.2. Characterization of Snf2 Family Proteins and Gene Structure

Physical and chemical properties of barley Snf2 proteins—theoretical isoelectric point (pI), molecular weight (MW), and grand average of hydropathicity (GRAVY)—were analyzed in the online ProParam software on the ExPASy server (https://web.expasy.org/protparam/ (accessed on 11 August 2022)). Subcellular localization was predicted on the Plant-mPloc server (http://www.csbio.sjtu.edu.cn/bioinf/plant-multi/ (accessed on 11 August 2022)). For domain organization analysis, all polypeptide sequences of identified barley Snf2 proteins were submitted to NCBI’s CDD platform to predict conserved domains. The exon–intron structure of barley Snf2 family genes was analyzed on the Gene Structure Display Server (GSDS; http://gsds.gao-lab.org/ (accessed on 26 July 2022)) in a General Feature Format (GFF) file (Hv_Morex.pgsb.Jul2020.HC.gff3; [26]).

### 4.3. Chromosome Localization, Duplication, and Evolution of Snf2 Genes

The chromosome distribution of all identified Snf2 family genes was determined on the Morex genome sequence from a GFF file (Hv_Morex.pgsb.Jul2020.HC.gff3; [26]). A Circos plot [55] was used to visualize the physical position of each gene. Gene duplication analysis was performed by using local BLAST comparisons with coding sequences. The coding sequences of Arabidopsis and rice Snf2 genes were obtained as above for protein sequences. Gene duplication events were defined by following the criteria of [56]: the alignment sequence should cover >70% of the longer gene in length; the aligned region should have an identity of >70%; and only 1 duplication event is counted for tightly linked genes. The linked gene pairs were also displayed in a Circos diagram. The ratio of Ka (non-synonymous substitution) to Ks (synonymous substitution) of Snf2 family genes was calculated in the KaKs_Calculator package v. 2.0 by using the YN method [57]; Ka/Ks < 1 indicates purifying selection, Ka/Ks = 1 indicates neutral selection, and Ka/Ks > 1 indicates positive selection. The genomic protein sequences of the wild barley OUH602 assembly were download from the Barley Bioresource Database (http://viewer.shigen.info/barley/download.php (accessed on 12 July 2021)). The pairwise Ka/Ks ratio of each orthologous pair between OUH602 and Morex was also computed in KaKs_Calculator by using the YN method. For these calculations, the coding sequences were aligned in MAFFT v. 7.475 software [58] with the guidance of the corresponding alignment of protein sequences. Multiple alignment files of coding and protein sequences were constructed in PALNAL v. 14 software [59] and then imported into KaKs_Calculator.

### 4.4. Phylogenetic Reconstruction

The protein sequences of Arabidopsis and rice Snf2 family members used for phylogenetic analysis were collected from The Arabidopsis Information Resource (TAIR, http://www.arabidopsis.org/ (accessed on 6 May 2021)) and Rice Genome Annotation Project (RGAP; http://rice.uga.edu/ (accessed on 25 September 2021)), respectively. The full-length sequences of Snf2 proteins acquired from barley (Morex), Arabidopsis, and rice were aligned by the ClustalW algorithm with default parameters [60], as implemented in MEGA7 software [61]. The phylogenetic tree was reconstructed by the maximum likelihood algorithm with 1000 bootstrap replicates from the multiple sequence alignment file in IQ-TREE2 v. 2.1.3 software [62]. The best-fit model for tree construction was determined by ModelFinder [63] as selected automatically in IQ-TREE2. To validate the search results among barley accessions, we generated the phylogenetic tree by the neighbor-joining (NJ) method in the Clustal Omega (CLUSTALO) alignment program [64], including the Arabidopsis and rice Snf2 family members to confirm the subfamily to which barley proteins belong.

### 4.5. Population Genetics Analysis

The coding sequences of each Snf2 genes across twenty-one barley accessions were aligned using ClustalW in CLC sequence viewer v7.8.1 (www.qiagenbioinformatics.com (accessed on 11 December 2022)), respectively. The single nucleotide polymorphisms (SNPs) in Snf2 genes among barley accessions were identified according to multiple alignment files and extracted by using SNP-sites software [65]. SnpEff software was employed to assess effects on protein function resulting from nucleotide variations and outputs the VCF file [66]. To determine the evolutionary divergence, SNPs located within barley Snf2 genes were used for principal component analysis (PCA) and population structure analysis. Phylogenetic tree for Snf2 gene-associated SNPs were constructed based on the PCA result, and population structure was evaluated using STRUCTURE software (http://pritch.bsd.uchicago.edu/structure.html (accessed on 12 December 2022); [67]) with predefined K values (the putative number of genetic groups) ranging from 1 to 8. The most likely value of *K* was indicated by log probability of the data (LnP(D)) and an ad hoc statistic ΔK through the change rate of LnP(D) between successive *K* value [68].

### 4.6. Expression Profile Analysis

The public RNA-seq datasets of 14 tissue samples provided by the IPK barley BARLEX server (http://barlex.barleysequence.org (accessed on 29 October 2021); [25]) were used to explore the expression patterns of barley *Snf2* genes in different tissues and at different development stages, namely root tissues (ROO), seedling shoots (LEA), etiolated seedlings (ETI), epidermal strips (EPI), developing inflorescence tissues (INF), rachis (RAC), third internode of tillers (NOD), lemma (LEM), and lodicule (LOD) dissected from inflorescence, as well as developing grains at 5 and 15 days after anthesis (CAR), and senescing leaves (SEN). Gene expression levels were estimated in terms of fragments per kilobase of transcript per million fragments mapped reads (FPKM) and were averaged where replicated samples were available. In addition, several biotic and abiotic stress expression datasets were downloaded from the NCBI Sequence Read Archive (http://www.ncbi.nlm.nih.gov/sra (accessed on 12 December 2022)) database to investigate the expression profiles of these genes. All reads from above datasets were mapped to the Morex v3 genome and processed into FPKM values [26]. A heatmap for gene expression data was constructed in the R package pheatmap v. 1.0.12 from log_2_-transformed mean FPKM values, and genes were clustered according to their expression patterns in the heatmap.

## Figures and Tables

**Table 1:** Snf2 family genes in Arabidopsis, rice, and barley.

Group	Subfamily	*Arabidopsis thaliana*	*Oryza sativa*	*Hordeum vulgare*
Snf2-like	Snf2	*AtCHR3 (SYD, AT2G28290)*	*OsCHR720 (Os06g0255200)*	*HORVU.MOREX.r3.7HG0669610*
		*AtCHR2 (BRM, AT2G46020)*	*OsCHR707 (Os02g0114000, Os02g0114033)*	*HORVU.MOREX.r3.6HG0543700*
		*AtCHR12 (AT3G06010)*	*OsCHR719 (Os05g0144300)*	*HORVU.MOREX.r3.1HG0019730*
		*AtCHR23 (AT5G19310)*		
	Lsh	*AtCHR1 (DDM1, AT5G66750)*	*OsCHR741 (Os03g0722400*	*HORVU.MOREX.r3.2HG0184370*
			*OsCHR746 (Os09g0442700)*	*HORVU.MOREX.r3.4HG0338270*
				*HORVU.MOREX.r3.7HG0646770*
	Iswi	*AtCHR11 (AT3G06400)*	*OsCHR727 (Os05g0150300)*	*HORVU.MOREX.r3.1HG0022440*
		*AtCHR17 (AT5G18620)*	*OsCHR728 (Os01g0367900)*	*HORVU.MOREX.r3.3HG0230070*
	ALC1	*AtCHR10 (AT2G44980)*	*OsCHR711 (Os03g0101700)*	*HORVU.MOREX.r3.4HG0416240*
	Chd1	*AtCHR5 (AT2G13370)*	*OsCHR705 (Os07g0660200)*	*HORVU.MOREX.r3.2HG0116700*
	Mi-2	*AtCHR6 (PICKLE, AT2G25170)*	*OsCHR702 (Os06g0183800)*	*HORVU.MOREX.r3.7HG0658830*
		*AtCHR7 (AT4G31900)*	*OsCHR703 (Os01g0881000)*	*HORVU.MOREX.r3.3HG0307470*
		*AtCHR4 (AT5G44800)*	*OsCHR729 (Os07g0497100)*	*HORVU.MOREX.r3.2HG0143700*
Swr1-like	Swr1	*AtCHR13 (PIE1, AT3G12810)*	*OsCHR709 (Os02g0689800)*	*HORVU.MOREX.r3.7HG0748860*
	Ino80	*AtCHR21 (INO80, AT3G57300)*	*OsCHR732 (Os03g0352450, Os03g0352500)*	*HORVU.MOREX.r3.4HG0375120*
	Etl1	*AtCHR19 (AT2G02090)*	*OsCHR714 (Os04g0566100)*	*HORVU.MOREX.r3.2HG0187800*
Rad54-like	Rad54	*AtCHR25 (RAD54, AT3G19210)*	*OsCHR733 (Os02g0762800)*	*HORVU.MOREX.r3.6HG0617770*
	ATRX	*AtCHR20 (AT1G08600)*	*OsCHR717 (Os10g0457700)*	*HORVU.MOREX.r3.1HG0040050*
	DRD1	*AtCHR35 (DRD1, AT2G16390)*	*OsCHR722 (Os07g0692600)*	*HORVU.MOREX.r3.2HG0108640*
		*AtCHR34 (AT2G21450)*	*OsCHR730 (Os03g0165200, Os03g0165266)*	*HORVU.MOREX.r3.4HG0402720*
		*AtCHR31 (AT1G05490)*	*OsCHR740 (Os02g0650800)*	*HORVU.MOREX.r3.3HG0271560*
		*AtCHR40 (AT3G24340)*	*OsCHR742 (Os05g0392400)*	*HORVU.MOREX.r3.1HG0064430*
		*AtCHR38 (AT3G42670)*	*OsCHR736 (Os07g0434500)*	*HORVU.MOREX.r3.2HG0128530*
		*AtCHR42 (AT5G20420)*	*OsCHR737 (Os06g0255700)*	*HORVU.MOREX.r3.5HG0501840*
			*OsCHR743 (Os08g0243833, Os08g0243866)*	
Rad5/16-like	Rad5/16	*AtCHR22 (AT5G05130)*	*OsCHR724 (Os07g0642400)*	*HORVU.MOREX.r3.2HG0119710*
		*AtCHR29 (AT5G22750)*	*OsCHR710 (Os02g0527100)*	*HORVU.MOREX.r3.6HG0559070*
		*AtCHR32 (AT5G43530)*	*OsCHR735 (Os04g0177300)*	*HORVU.MOREX.r3.2HG0103780*
		*AtCHR37 (AT1G05120)*	*OsCHR731 (Os07g0511500)*	*HORVU.MOREX.r3.2HG0141740*
		*AtCHR41 (AT1G02670)*		
	Ris1	*AtCHR26 (AT3G16600)*	*OsCHR706 (Os01g0779400)*	*HORVU.MOREX.r3.3HG0293510*
		*AtCHR27 (AT3G20010)*	*OsCHR715 (Os04g0629300)*	*HORVU.MOREX.r3.2HG0199060*
		*AtCHR28 (AT1G50410)*	*OsCHR725 (Os08g0180300)*	*HORVU.MOREX.r3.7HG0696030*
		*AtCHR30 (AT1G11100)*		
		*AtCHR33 (AT1G61140)*		
	SHPRH	*AtCHR39 (AT3G54460)*	*OsCHR708 (Os01g0952200)*	*HORVU.MOREX.r3.3HG0320710*
		*AtCHR36 (AT2G40770)*	*OsCHR739 (Os07g0680500)*	
SSO1653-like	Mot1	*AtCHR16 (AT3G54280)*	*OsCHR701 (Os02g0161400)*	*HORVU.MOREX.r3.1HG0094930*
	ERCC6	*AtCHR8 (AT2G18760)*	*OsCHR704 (Os01g0102800)*	*HORVU.MOREX.r3.3HG0231890*
		*AtCHR9 (AT1G03750)*	*OsCHR713 (Os05g0247900)*	*HORVU.MOREX.r3.2HG0132190*
		*AtCHR24 (AT5G63950)*	*OsCHR712 (Os04g0692700)*	*HORVU.MOREX.r3.2HG0217790*
			*OsCHR745 (Os01g0636700)*	*HORVU.MOREX.r3.6HG0559010*
SMARCAL1-like	SMARCAL1	*AtCHR14 (AT5G07810)*	*OsCHR726 (Os07g0598300)*	*HORVU.MOREX.r3.2HG0128410*
		*AtCHR18 (AT1G48310)*	*OsCHR721 (Os07g0636200)*	

Snf2, sucrose nonfermenting 2; Lsh, lymphoid-specific helicase; Iswi, imitation switch; ALC1, amplified in liver cancer 1; Chd1, chromodomain helicase DNA-binding 1; Mi-2, multi-subunit chromatin remodeling protein 2; Swr1, SWI2/SNF2-related 1, Ino80, inositol requiring 80; Etl1, enhancer trap locus 1; Rad54, recombination and DNA repair protein 54; ATRX, X-linked mental retardation with alpha-thalassemia; DRD1, defective in RNA-directed DNA methylation 1; Rad5/16, recombination and DNA repair protein 5/16; Ris1, role in silencing protein 1; SHPRH, Snf2 histone linker PHD RING helicase; Mot1, modifier of transcription 1; ERCC6, excision repair chromatin remodeling factor 6; SMARCAL1, SWI/SNF-related matrix-associated actin-dependent regulator of chromatin, subfamily A like 1.

**Table 2:** Distribution of Snf2 genes within 18 subfamilies among cultivated and wild barley genome assemblies.

Accession	Total	Snf2	Lsh	Iswi	ALC1	Chd1	Mi-2	Swr1	Ino80	Etl1	Rad54	ATRX	DRD1	Rad5/16	Ris1	SHPRH	Mot1	ERCC6	SMARCAL1
Akashinriki ^a^	41	3	3	2	1	1	3	1	1	1	1	1	7	4	3	1	1	5	2
Barke ^a^	41	3	3	2	1	1	3	1	1	1	1	1	7	4	3	1	1	5	2
Golden_Promise ^a^	40	3	3	2	0	1	3	1	1	1	1	1	7	4	3	1	1	5	2
Hockett ^a^	39	3	2	2	1	1	2	1	1	1	1	1	7	4	3	1	1	5	2
HOR_10350 ^a^	40	3	3	2	1	1	3	1	1	1	1	1	7	4	3	1	1	4	2
HOR_13821 ^a^	39	3	3	2	1	1	2	1	1	1	1	1	7	4	3	1	1	4	2
HOR_13942 ^a^	40	3	3	2	1	1	3	1	1	1	1	1	6	4	3	1	1	5	2
HOR_21599 ^a^	39	3	3	2	1	1	3	1	1	1	1	1	7	4	3	1	1	3	2
HOR_3081 ^a^	41	3	3	2	1	1	3	1	1	1	1	1	7	4	3	1	1	5	2
HOR_3365 ^a^	38	3	2	2	1	1	3	1	1	1	1	1	7	4	3	1	1	3	2
HOR_7552 ^a^	39	3	3	2	1	1	3	1	1	1	1	1	7	4	3	1	1	3	2
HOR_8148 ^a^	41	3	3	2	1	1	3	1	1	1	1	1	7	4	3	1	1	5	2
HOR_9043 ^a^	40	3	2	2	1	1	3	1	1	1	1	1	7	4	3	1	1	5	2
Igri ^a^	41	3	3	2	1	1	3	1	1	1	1	1	7	4	3	1	1	5	2
OUN333 ^a^	41	3	3	2	1	1	3	1	1	1	1	1	7	4	3	1	1	5	2
RGT_Planet ^a^	41	3	3	2	1	1	3	1	1	1	1	1	7	4	3	1	1	5	2
ZDM01467 ^a^	39	3	3	2	1	1	3	1	1	1	1	1	7	4	2	1	1	4	2
ZDM02064 ^a^	39	2	2	2	1	1	3	1	1	1	1	1	7	4	3	1	1	5	2
B1K-04-12 ^b^	40	3	3	2	1	1	3	1	1	1	1	1	7	4	3	1	1	4	2
OUH602 ^b^	40	3	3	2	1	1	3	1	1	1	1	1	7	4	3	1	1	4	2

^a^ Cultivated barley. ^b^ Wild barley.

## Data Availability

All data supporting the findings of this study are available within this article and the Supplementary Materials published online.

## Supplementary Materials

The following supporting information can be downloaded at: https://www.mdpi.com/article/10.3390/ijms24010457/s1.
