# Supplementary material for: Genome-Wide Analysis of Snf2 Gene Family Reveals Potential Role in Regulation of Spike Development in Barley
Source: Int J Mol Sci. 2022 Dec 27;24(1):457. doi: 10.3390/ijms24010457 (PMC9820626; doi:10.3390/ijms24010457)
Supplement: Supplementary file 1 [file ijms-24-00457-s001.zip › Supplementary figures.pdf]

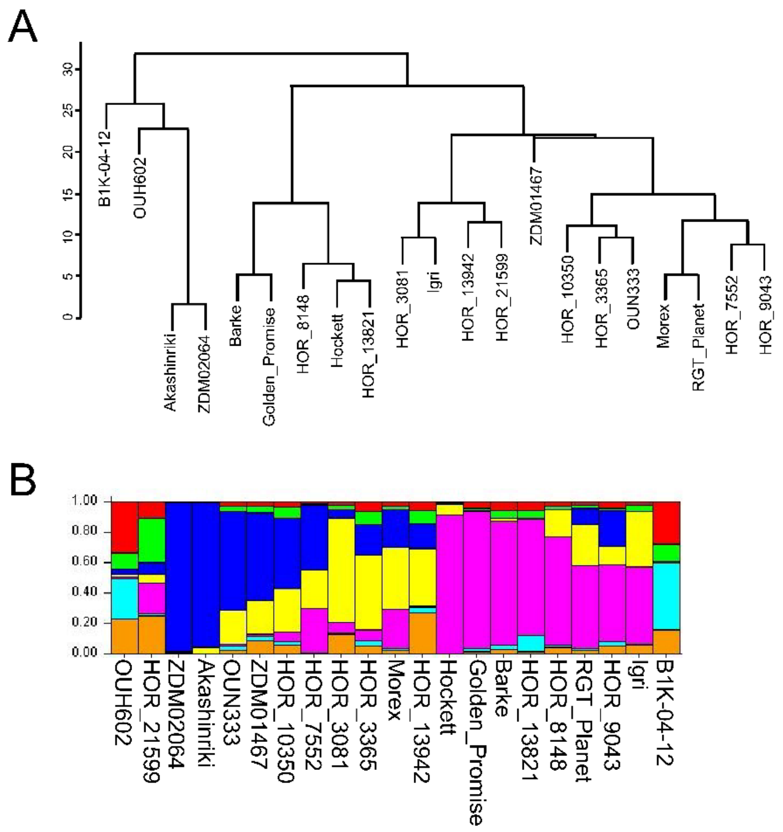

Figure S1. Population structure analysis of the Snf2-associated SNPs among different barley accessions. (A) Cluster dendrogram of SNPs across barley accessions. (B) Population structure with K = 7.

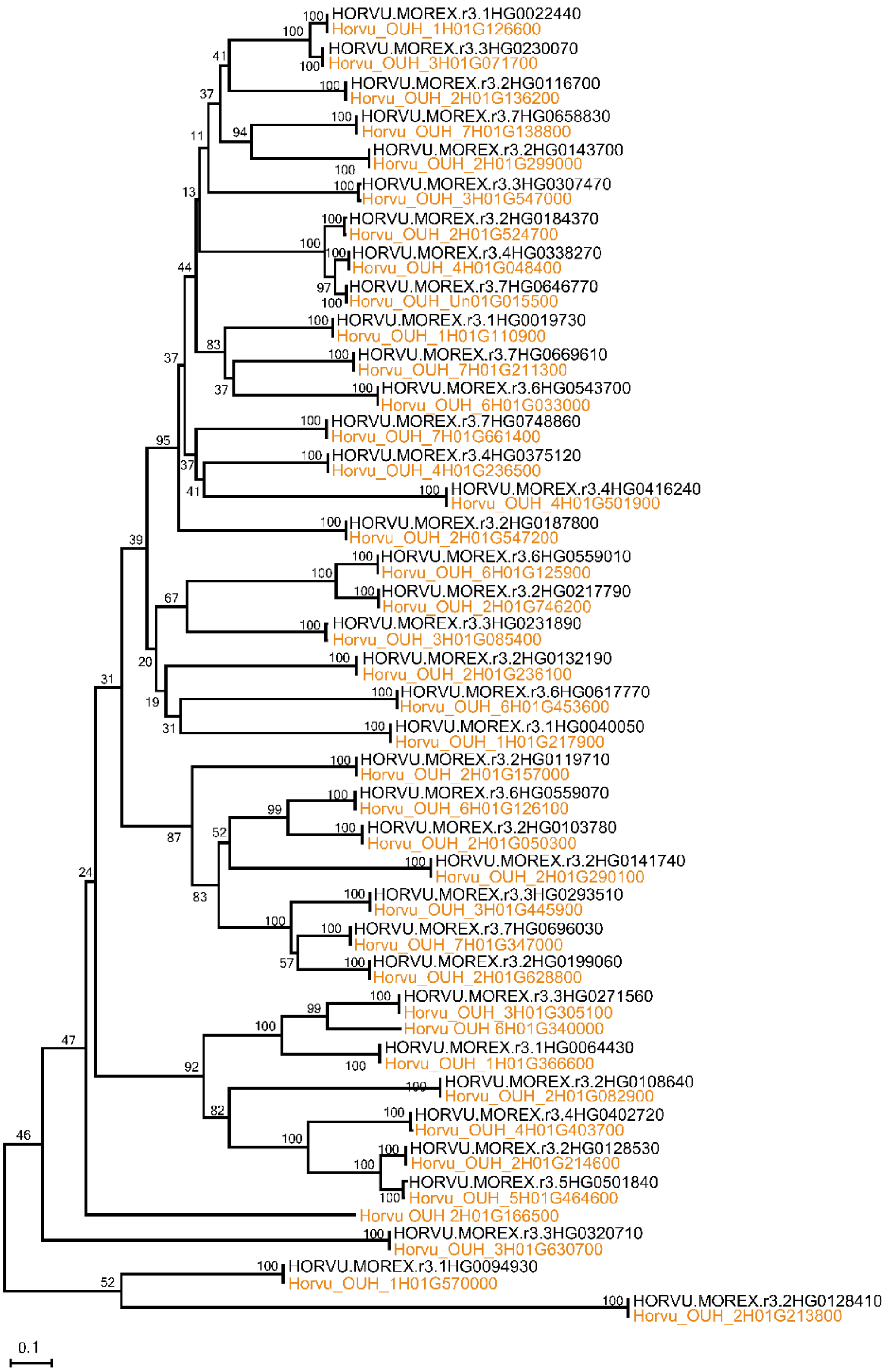

Figure S2. Phylogeny of Snf2 family proteins from wild (OUH602, orange text) and cultivated (Morex, black text) barley. The neighbor-joining tree was constructed with CLUSTALW in MEGA7 software on the basis of an alignment of full-length amino acid sequences. Bootstrap probabilities are shown at each branch node.
